# A Rare Case of Small Bowel Obstruction Secondary to Pelvic Inflammatory Disease

**DOI:** 10.7759/cureus.13983

**Published:** 2021-03-18

**Authors:** Ahmed K Ahmed, Ahamed M Elkhair, Omeralfaroug Adam, Mohamedanwar Ghandour

**Affiliations:** 1 Radiology, Countess of Chester Hospital, Chester, GBR; 2 Internal Medicine, University of Medical Sciences and Technology, Khartoum, SDN; 3 Internal Medicine, Wayne State University Detroit Medical Center, Detroit, USA; 4 Internal Medicine/Nephrology, Wayne State University Detroit Medical Center, Detroit, USA

**Keywords:** bowel obstruction, pelvic inflammatory disease

## Abstract

Small bowel obstruction (SBO) secondary to pelvic inflammatory disease (PID) is a rare complication only reported on a few occasions. We presented a 38-year-old female with an acute abdomen secondary to PID diagnosed via CT and MRI abdomen. The patient was treated in a conservative manner and recovered with no further complications. In our case, the learning point is the consideration of such an etiology in women with no previous surgical history presenting with an acute abdomen.

## Introduction

Pelvic inflammatory disease (PID) is an infection of the female upper genital tract [1]. It is vital to diagnose and treat PID swiftly to prevent complications, including chronic pelvic pain, infertility, and ectopic pregnancy [2]. The risk of recurrence is more likely seen in adolescents as compared to adult females [3]. However, many PID cases are sexually transmitted, with fewer than 15% of acute PID cases associated with enteric or respiratory pathogens that have colonized the genital tract [4].

IUD placement is the most commonly used method of reversible contraception globally and is used by an average of 23% of females using contraception, with a range of <2% to >40% based on the country [5]. Risk factors leading to complications, such as PID, include young age, breastfeeding, and postpartum/post-abortion placement [6].

On the other hand, small bowel obstruction (SBO) is a common surgical emergency defined as the disturbance of the normal flow of intraluminal content secondary to either mechanical or functional factors [7,8] This leads to the dilation of the bowel, which causes an edematous alteration of the lumen. As a result, the bowel's normal function is lost, in addition to complications such as infections occur [9]. Local peritonitis influences the normal function of the small or large bowel. This condition may be diagnosed by clinical examination of the abdominal-hypogastric area, by laboratory and by imaging data. Conditions with these abscesses, and extended local inflammation are difficult in surgery with postoperative complications. More conservative treatment such as antibiotics or minimally invasive radiologic techniques under CT guidance are important for the final treatment.

Also, acute SBO accounts for 2% to 4% of emergency department visits, with 15% of admissions and 20% of emergency surgical operations for abdominal pain [10,11]. We report a 38-year-old lady presenting with SBO secondary to PID, an etiology leading to bowel obstruction.

## Case presentation

**Figure 1:**
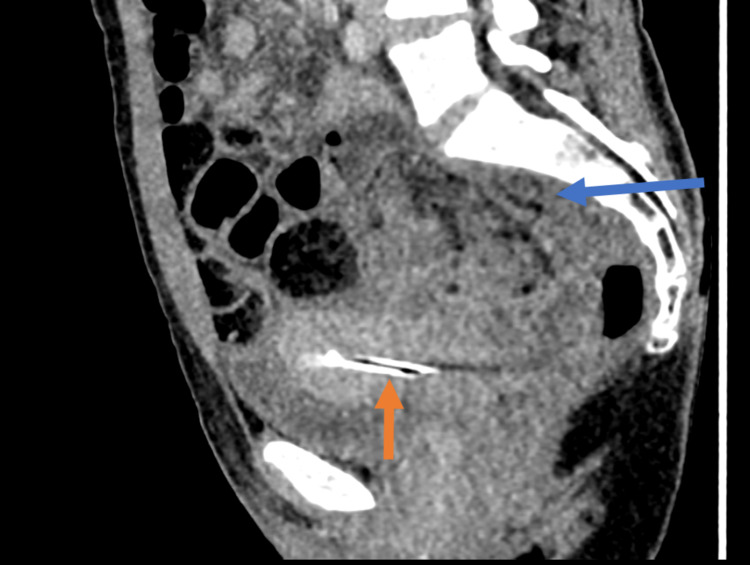
Sagittal portal venous phase CT of the abdomen and pelvis. Note the IUD in situ (orange arrow) and the presacral fat stranding (blue arrow). IUD: intrauterine device.

**Figure 2:**
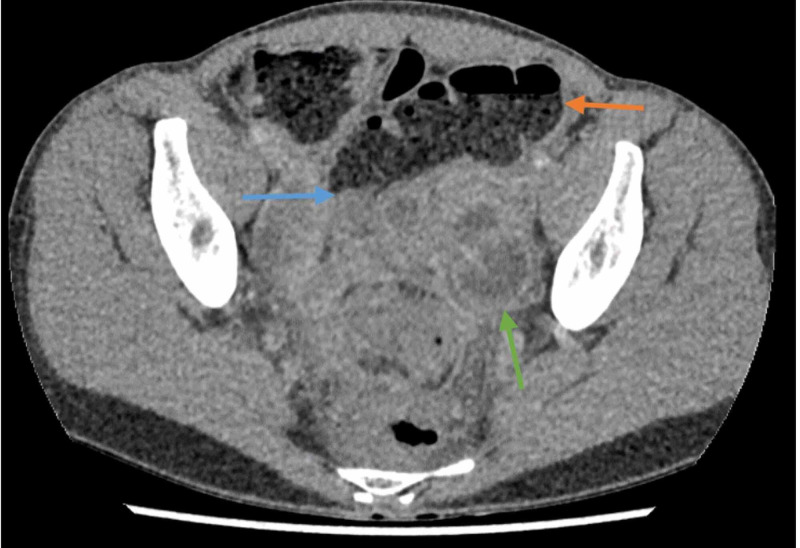
Axial portal venous phase CT of the abdomen and pelvis. Note the transition point (blue arrow) causing proximal dilatation of the sigmoid colon. There are multiple regions of low signal with enhancing rims, representing abscesses (green arrow).

**Figure 3:**
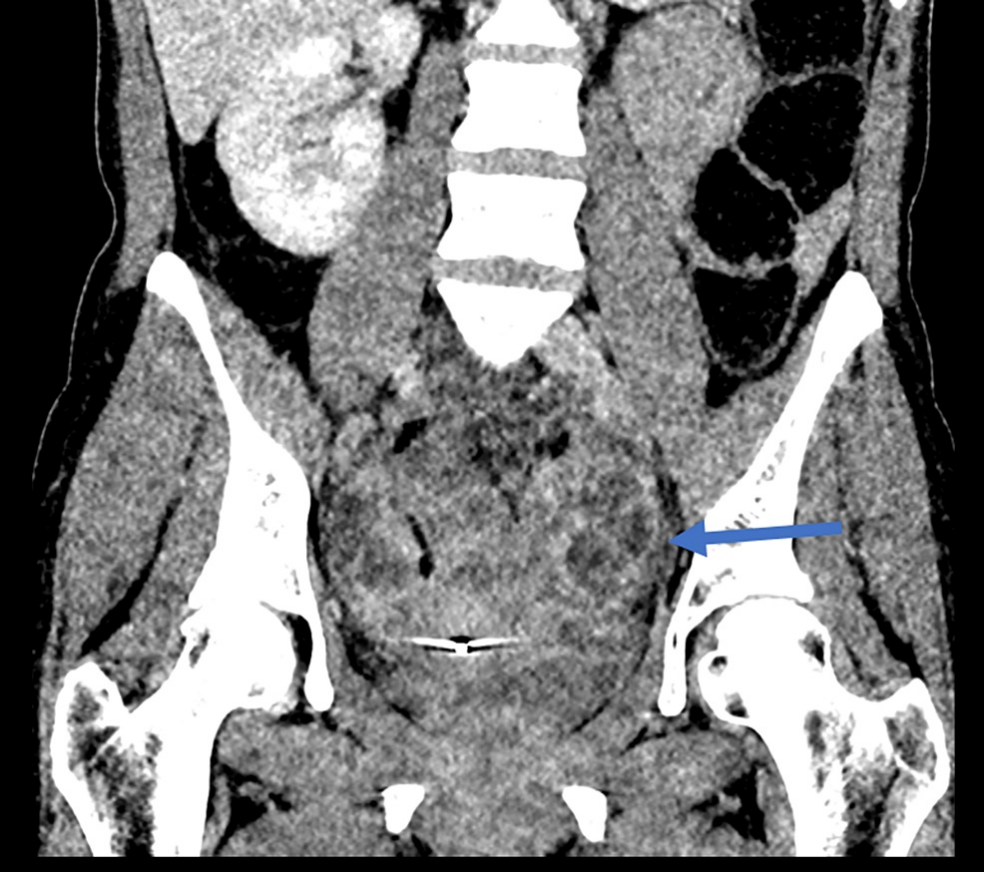
Coronal view of CT of the abdomen and pelvis. Blue arrow showing coronal view of abscess.

A 38-year-old female with a past medical history of menorrhagia and post-coital bleeding presented with severe abdominal pain. This began one day prior to admission and was associated with a three-week history of constipation. The patient did not have a significant surgical or gynecological history. However, she is sexually active and using an intrauterine device (IUD) for protection. Physical exam showed normal vital signs, and the abdominal exam revealed diffuse distention with hyperactive bowel sounds with no signs of peritonitis. Blood workup was remarkable for an elevated WBC of 18.9. Otherwise, liver and renal function tests, erythrocyte sedimentation rate, and C-reactive protein levels were normal. Plain abdominal X-ray findings were consistent with subacute SBO. A CT abdomen was performed showing dilatation of the large bowel with a transition point in the sigmoid colon. Distal to this there was thickening of the distal sigmoid colon and rectum with surrounding mesorectal and presacral fat stranding. Multiple bilateral pelvic cystic lesions were identified which were suspicious for abscesses (Figures 1-3). 

**Figure 4:**
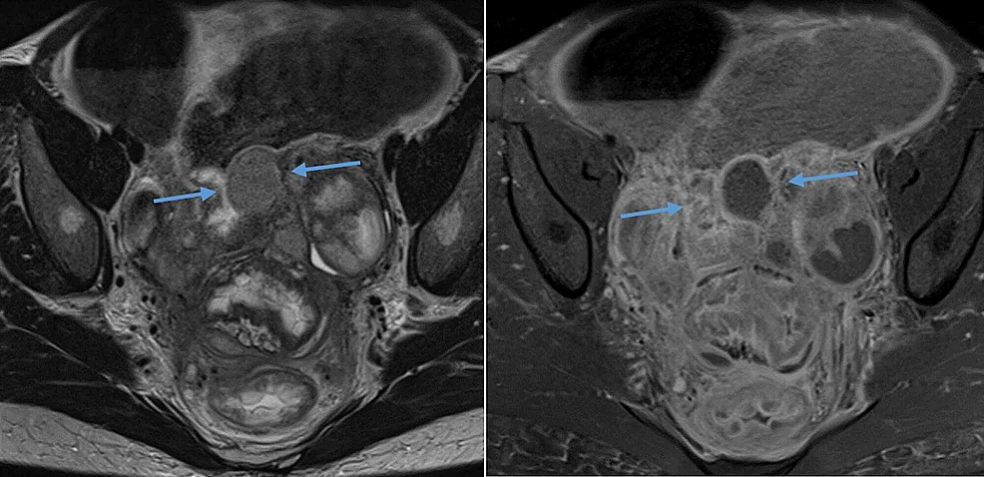
Axial T2 weighted and contrast-enhanced T1 weighted MRI pelvis showing the multiple pelvic abscesses and left-sided tubo-ovarian abscess. Note the position of one of the abscesses adjacent to the transition point in the colon (blue arrows). The distal sigmoid colon loops are thickened and inflamed, while the proximal loops are distended.

**Figure 5:**
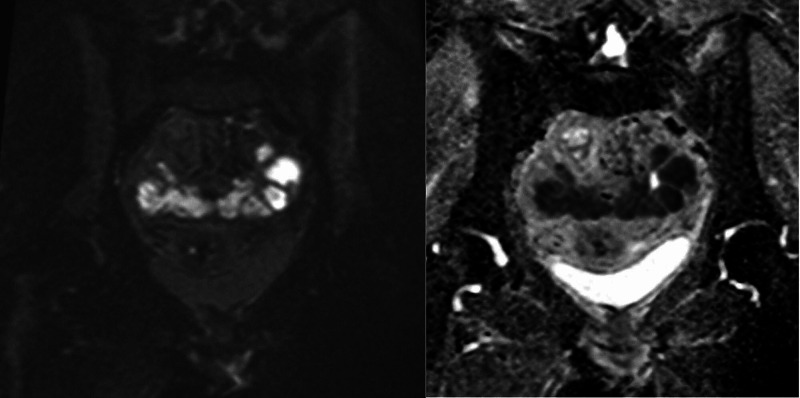
Coronal b800 diffusion weighted and apparent diffusion coefficient MRI pelvis showing high signal within the pelvic abscesses with signal drop out indicating restricted diffusion.

A pelvic MRI was performed which showed extensive pelvic inflammation. This was complicated by multiple pelvic collections which showed high signal on T2, rim enhancement and restricted diffusion in keeping with abscesses (the largest was anterior to the rectum and measured 3.5 x 2.5cm). The fallopian tubes were distended and enhancing, indicating pyosalpinx, with evidence of a tubo-ovarian abscess on the left side. The rectum and sigmoid were thickened and enhancing, with dilatation of the proximal sigmoid in keeping with bowel obstruction. Adjacent to the transition point was an abscess, which was inseparable from the mural surface. Incidental note was made of a small endometrioma in the right ovary, with normal appearance of the bladder, uterus, and cervix (Figures 4, 5). 

**Figure 6:**
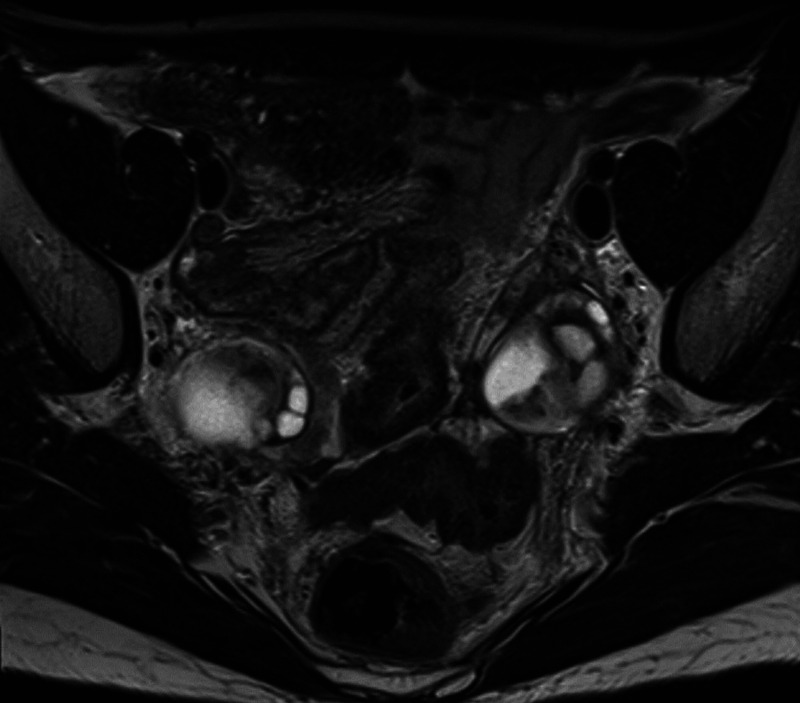
Axial T2 weighted MRI pelvis showing resolution of the pelvic abscesses and improvement in the bowel wall inflammatory change. The ovaries are cystic bilaterally.

The patient was treated conservatively with nil by mouth, intravenous (IV) fluids, and nasogastric tube (NGT) decompression. The IUD was removed and a culture was done which showed a negative result. Broad-spectrum antibiotic therapy (doxycycline and ceftriaxone) was given. As the patient was clinically well, she was discharged in a stable condition and a repeat MRI was completed as an outpatient after completing her course. This showed resolution of the pelvic abscesses, with improvement in the sigmoid colon and rectal. Minor residual diffuse soft tissue enhancement remained (Figure 6).

## Discussion

We presented a 38-year-old lady presenting with SBO secondary to PID, a rare etiology leading to bowel obstruction. This case has been scarcely described in the past, with cases reported on only a few occasions [12,13]. CT abdomen with IV contrast is the imaging modality for all patients with suspicion of bowel obstruction If no contraindications are detected [14]. CT abdomen findings of the reported case revealed the large bowel's dilatation with a transition point in the sigmoid colon, consistent with SBO. Also, there was thickening of the distal sigmoid colon and rectum surrounding mesorectal and presacral fat stranding. These findings were similar to other previous cases with an etiology of PID [12].

Furthermore, an MRI of the abdomen showed fallopian tubes were distended and enhancing, indicating pyosalpinx, with evidence of a tubo-ovarian abscess on the left side. The rectum and sigmoid were thickened and enhancing, with dilatation of the proximal sigmoid in keeping with bowel obstruction. While imaging has minimal usage in the diagnosis of PID, there is usefulness in assessing complications related to it [15,16].

Our patient was treated conservatively, the IUD was removed, and broad-spectrum antibiotic therapy (doxycycline and ceftriaxone) was given. Non-surgical treatment has shown to be an acceptable form of therapy with an overall success rate of 65% to 80% [17]. The antibiotic therapy of choice is also consistent with the previous literature [18]. For Patients with severe infection, a carbapenem (imipenem, meropenem, or doripenem) should be chosen as per the literature [6]. However, our patient did not show any indication of a severe form of an infection, resulting in not including this into our management plan.

## Conclusions

In summary, SBO secondary to PID is a rare but significant complication. It should be considered in females of reproductive age with no significant surgical history presenting with an acute abdomen.
